# CAPTCHA as a Visual Performance Metric in Active Macular Disease

**DOI:** 10.1155/2019/6710754

**Published:** 2019-06-09

**Authors:** Gautam Vangipuram, Aaron Y. Lee, Kasra A. Rezaei, Lisa C. Olmos De Koo, Yewlin E. Chee, Jennifer R. Chao, Catherine Egan, Cecilia S. Lee

**Affiliations:** ^1^Department of Ophthalmology, University of Washington, Seattle, WA 98104, USA; ^2^Moorfields Eye Hospital, London, UK

## Abstract

**Purpose:**

CAPTCHA (completely automated public turing test to tell computers and humans apart) was designed as a spam prevention test. In patients with visual impairment, completion of this task has been assumed to be difficult; but to date, no study has proven this to be true. As visual function is not well measured by Snellen visual acuity (VA) alone, we theorized that CAPTCHA performance may provide additional information on macular disease-related visual dysfunction.

**Methods:**

This was designed as a pilot study. Active disease was defined as the presence of either intraretinal fluid (IRF) or subretinal fluid (SRF) on spectral-domain optical coherence tomography. CAPTCHA performance was tested using 10 prompts. In addition, near and distance VA, contrast sensitivity, and reading speed were measured. Visual acuity matched pseudophakic patients were used as controls. Primary outcome measures were average edit distance and percent of correct responses.

**Results:**

70 patients were recruited: 33 with active macular disease and 37 control subjects. Contrast sensitivity was found to be significantly different in both the IRF (*p* < 0.01) and SRF groups (*p* < 0.01). No significant difference was found comparing the odds ratio of average edit distance of active disease (IRF, SRF) vs. control (OR 1.09 (0.62, 1.90), 1.10 (0.58, 2.05), *p*=0.77,  0.77) or percent correct responses of active disease vs. control (OR 0.98 (0.96, 1.01), 1.09 (0.58, 2.05), *p*=0.22, 0.51) in CAPTCHA testing. The goodness of fit using logistic regression analysis for the dependent variables of either IRF or SRF did not improve accounting for average edit distance (*p*=0.49,  *p*=0.27) or percent correct (*p*=0.89,  *p*=0.61).

**Conclusions:**

Distance VA and contrast sensitivity are positively correlated with the presence of IRF and SRF in active macular disease. CAPTCHA performance did not appear to be a significant predictor of either IRF or SRF in our pilot study.

## 1. Introduction

**Figure 1:**
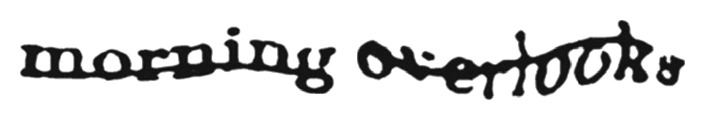
Example of CAPTCHA prompt presented as challenge task to users. To pass the test, a user must enter the correct alphanumeric sequence presented as displayed.

Computer automated public turing test to tell computers and humans apart (CAPTCHA) has gained popularity in recent years as an electronic visual challenge-response test of human authenticity (Figure 1). The test involves a user correctly entering all alphanumeric symbols displayed in a text prompt. Although CAPTCHA testing is useful in thwarting potential hackers and spamming, passing it can prove challenging to those with visual impairment [1, 2]. Responders with diseases affecting central vision, in particular the macula, may have even more difficulty completing these tasks. To this end, quantifying patients' level of performance on CAPTCHA testing may provide a noninvasive, sensitive, and easy way to perform method of potentially monitoring active macular disease.

To evaluate macular disease, CAPTCHA performance may provide an advantage over other visual acuity metrics methods in assessing central visual acuity. Traditionally, Snellen visual acuity is the standard metric for assessing vision and treatment outcomes [3–9]. Nonetheless, Snellen testing often fails to address aspects of important visual impairment to patients for daily functioning and quality of life. Elliott et al. reported on a series of thirty-three patients with a history of visually significant cataract in at least one eye. The authors measured each patient's visual acuity, brightness acuity, and binocular contrast sensitivity and correlated these findings to a questionnaire of vision-related quality of life [10]. Binocular contrast sensitivity correlated more strongly to visual quality of life than Snellen visual acuity alone. Similarly, Hazel et al. found that low-contrast visual acuity, contrast sensitivity, and binocular reading speed had the strongest correlation with visual quality of life in 28 patients with active macular disease from either presumed ocular histoplasmosis syndrome (POHS) or exudative macular degeneration [11].

Snellen visual acuity also fails to address visual symptoms in individuals with noncenter involving macular disease. Quantifying a patient's CAPTCHA performance may provide a functional endpoint that is noninvasive, inexpensive, and easy to perform compared to structural makers of disease activity such as optical coherence tomography (OCT) and optical coherence tomography angiogram (OCTA). To our knowledge, CAPTCHA performance has not been studied in patients with visual disability, and this is the first study to examine CAPTCHA performance in patients with active macular disease [12–20]. Our study hypothesized that patients with macular disease, defined as active diabetic macular edema (DME) or exudative age-related macular degeneration (AMD), would perform poorly on a series of CAPTCHA prompts compared to visually matched pseudophakic controls.

## 2. Materials and Methods

The study was a prospective cohort study. Patients were recruited from the University of Washington Eye Institute, Seattle, WA. Institutional review board approval was obtained, and the study followed the World Medical Association Declaration of Helsinki Ethical Principles for medical research involving human subjects. Inclusion criteria for the macular disease cohort were defined as having ICD-10 code diagnoses of diabetic macular edema (E11.311) or exudative age-related macular degeneration (H35.32), OCT confirmed intraretinal fluid (IRF) or subretinal fluid (SRF), and English as the primary spoken language. Exclusion criteria for the macular disease cohort were defined as patients with concomitant pathology affecting the central visual axis (e.g., visually significant cataract, central corneal scar, and amblyopia), best near corrected visual acuity worse than 20/50, and the presence of both IRF and SRF. Control patients were pseudophakic, spoke English as their primary language, and had a best-corrected visual acuity of at least 20/20 with no other diseases affecting the central visual axis. Only one eye from each patient was tested. If both eyes met inclusion and exclusion criteria, a coin flip was used to determine the testing eye.

All patients were asked to complete a series of visual tasks. Control patients were blurred to a predetermined visual acuity (20/20–20/50) using positive power spherical lenses in an attempt to create a 1 : 1 match of visual acuity to the macular disease cohort. Distance acuity was tested using a standard Snellen chart while near distance was tested using a Rosenbaum near point acuity card at 14 inches. CAPTCHA testing was performed at the same near distance, 14 inches, as the Rosenbaum near point test by using an iPAD Air (Model A1566, Apple Inc. Cupertino, CA) tablet. A series of 10 CAPTCHA images were presented to each patient in a randomized order. Typed responses were recorded in a separate database. Contrast sensitivity (logcontrast) was tested using a Pelli–Robson chart. Reading speed in seconds was tested at near distance using a 156-word paragraph.

The primary outcome measure was average edit distance. In computational science, edit distance is defined as the minimum number of operations needed to transform one string of letters into another. The average edit distance of each subject's responses to the ten randomized CAPTCHA prompts was calculated. The secondary outcome measure was percent correct. For example, if a patient answered all of one CAPTCHA prompt exactly as displayed, this was marked as correct, and any deviation from a prompt was marked incorrect. Thus, if a patient answered five CAPTCHA prompts correctly out of ten, the value of this outcome variable would be 50%.

### 2.1. Statistical Methods

All statistical analyses were completed using SPSS version 24 (IBM corporation, New York, United States) and independently verified by a biostatistician. Logistic regression analysis was used to determine the goodness of fit (Nagelkerke *R*^2^) of a base model consisting of the dependent variable (IRF or SRF) and covariates (visual acuity, reading speed, and contrast sensitivity). Average edit distance and percent correct were independently added to this base model, and chi-square analysis was used to determine significance in model fit.

## 3. Results

Seventy patients were recruited for this study (Table 1). Thirty-three patients had active macular disease comprising either subretinal fluid or intraretinal fluid. Thirty-seven patients were recruited as controls. Of the active disease cohorts, 14 (42.4%) had DME while 19 (57.6%) had AMD. There was no statistical difference in age (*p*=0.552), gender (*p*=0.824), or race (*p*=0.214) between the active disease and control groups. When near visual acuity was stratified into 5 groups (20/20, 20/25, 20/30, 20/40, and 20/50), no significant difference was found between active disease and control cohorts.

**Figure 2:**
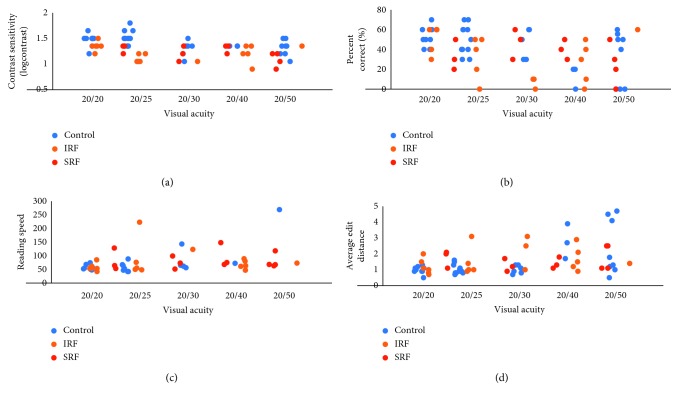
Scatter plot for contrast sensitivity (a), percent correct (b), reading speed, (c) and average edit distance (d) vs. visual acuity.

The active disease cohort was further stratified into patients with IRF (*n*=20) and SRF (*n*=13). On univariate analysis, there was no significant difference in average edit distance between either the IRF group or SRF group vs. control (Table 2). Likewise, no significant difference was found in percent correct between either the IRF group or SRF group vs. control. As demonstrated in Figure 2, no distinct trend was seen in comparing the IRF group or SRF group vs. control for average edit distance, percent correct, or reading speed. However, contrast sensitivity performance was noted to significantly differ between both IRF vs. control (*p*=0.016) and SRF vs. control (*p*=0.024).

Logistic regression analysis is shown for both IRF and SRF cohorts (Table 3). The base model (A or B) assumes either IRF (A) or SRF (B) as the dependent variable with covariates of visual acuity, reading speed, and contrast sensitivity. The −2  log likelihood, which can be interpreted as a pseudo *R*^2^ for this model, was calculated. For both the IRF and SRF base models, covariates of average edit distance and percent correct were added to determine if this improved the goodness of fit of the model. In neither IRF (*x*^2^ = 0.07, *p*=0.79) nor SRF (*x*^2^ = 0.15, *p*=0.70), did the addition of average edit distance improve the goodness of fit of the model. A similar result was found when the secondary outcome variable of percent correct was added to the model: IRF (*x*^2^ = 0.68, *p*=0.410) and SRF (*x*^2^ = 0.02, *p*=0.88).

## 4. Discussion

This was a prospective cohort study to determine whether macular intraretinal or subretinal fluid correlated with CAPTCHA performance. The “goodness of fit” of the logistic regression model predicting either IRF or SRF did not improve when average edit distance was added to the base model. Similarly, no improvement was seen in comparing percent correct to its respective base model. These results likely suggest that CAPTCHA performance is not an independent predictor of intraretinal or subretinal fluid in active macular disease. Our findings show that patients with macular disease perform poorly on contrast sensitivity assessment in spite of relatively preserved central visual acuity as previously reported [21]. Both the IRF and SRF cohorts performed significantly worse on contrast testing compared to pseudophakic visual acuity-matched controls.

CAPTCHA images contain a series of variably separated optotypes that create a challenging visual prompt to the examiner. Although CAPTCHA images do not fit a standardized visual testing method, it mostly resembles ascertaining a subject's minimum legible acuity. Both Snellen distance and Rosenbaum near point testing also assess a subject's minimum legible acuity by differentiating increasingly smaller optotypes to determine a final visual acuity [22]. Unlike these tests, however, CAPTCHA does not employ strong serifs in its font, making its text more difficult to discern. Furthermore, given the variation in CAPTCHA letter size within each image, it is difficult to determine the equivalent visual acuity with Snellen testing. However, the majority of letters in each test image were between 10- and 14-point font or 20/70 and 20/100 Snellen equivalent.

In addition, all CAPTCHA prompts in our study employed high contrast, black print on white background. A tablet computer was chosen to administer the CAPTCHA prompts to most realistically simulate real-life testing conditions and facilitate a standardized near acuity. However, the increased background luminance during testing has been shown to improve a subject's visual acuity and small letter contrast sensitivity [23, 24]. Thus, the heightened background luminance of the tablet may have aided the subject compared to the standardized photopic room testing conditions (90 lux) of the Pelli–Robson examination. In addition, the enhanced contrast of CAPTCHA images may be above the threshold of how much intraretinal or subretinal pathologies affects one's visual function, which could explain why CAPTCHA performance was not significantly different in patients with active disease versus control subjects.

The exact pathophysiology of decreased contrast sensitivity has not been fully elucidated. Poor performance on contrast sensitivity testing is seen in a variety of diseases including glaucoma, optic neuritis, and macular degeneration [21]. Arend et al. reported a series of 20 patients with diabetes mellitus without clinically significant macular edema with better than 20/25 vision. The authors found a significant correlation between foveal avascular zone area and contrast sensitivity, suggesting parafoveal capillary dropout may have contributed to a decrease in contrast sensitivity [25]. García et al. performed a retrospective study on 62 multiple sclerosis patients and found a significant correlation with optic nerve disease activity and contrast sensitivity performance. Our study adds to the existing literature demonstrating poor contrast delineation in patients with intraretinal fluid or subretinal fluid [26, 27].

The strengths of this study are its prospective nature, which allowed for well-defined inclusion and exclusion criteria. In addition, OCT images obtained at the time of testing allowed for determination of disease activity in real time. Limitations include the lack of quantification of center vs. noncenter, involving fluid on OCT imaging and the absence of OCT images from pseudophakic controls.

Additionally, the technique of visual blurring using positive convex lenses has been described in the literature to assess its effect on the completion of visual tasks and the comprehension of visual cues [28, 29]. Although visual blurring effectively matched the control population's visual acuity to the disease cohort, this technique is only meant to simulate retinal pathology and does not entirely mimic the pathology seen in DME and AMD.

In summary, visual function tests other than Snellen visual acuity are important in evaluating ocular diseases. In particular, contrast sensitivity should be considered in the clinical evaluation of active macular disease. Although visually impaired patients can struggle with CAPTCHA task completion, our study results show that neither intraretinal fluid nor subretinal fluid correlate with worsened CAPTCHA performance. We would expect, therefore, that using CAPTCHA to prevent hacking attempts would not exclude patients affected with active macular disease if their visual acuity allowed them to perform the test adequately. Further studies are needed to determine if CAPTCHA performance correlates to other structural markers of ocular pathology.

## 5. Conclusions

CAPTCHA performance is not a significant predictor of intraretinal fluid or subretinal fluid in patients with active macular disease. Ancillary tests of visual function, particularly contrast sensitivity, correlate to macular disease. Further studies are needed to determine whether CAPTCHA performance is associated with other features of macular diseases.

## Figures and Tables

**Table 1:** Baseline demographics and clinical characteristics for active disease (either intraretinal fluid or subretinal fluid) and pseudophakic control patients.

	Active disease (*n*=33)	Control (*n*=37)	*p* value
	Mean ± Std. dev.	Mean ± Std. dev.	
*Age*	69.2 ± 15.0	69.7 ± 8.0	0.55

*Gender*			
Female	21 (47.7%)	23 (52.3%)	0.82
Male	12 (53.3%)	14 (46.7%)	

*Race*			
White	25 (75.7%)	30 (81.1%)	0.21
Black	2 (6.1%)	2 (5.4%)	
Asian	0 (0%)	3 (8.1%)	
Hispanic	6 (18.1%)	2 (5.4%)	

*Visual acuity group*			
1 (20/20)	6 (18.2%)	10 (27.0%)	0.83
2 (20/25)	7 (21.2%)	10 (27.0%)	
3 (20/30)	6 (18.2%)	6 (16.2%)	
4 (20/40)	9 (27.2%)	3 (8.1%)	
5 (20/50)	5 (15.2%)	8 (21.6%)	

*BCVA near logMAR*	0.19 ± 0.14	0.17 ± 0.15	0.65

*Disease*			
DME	14 (42.4%)	—	—
AMD	19 (57.6%)	—	—

No statistically significant difference was found between active disease and control cohort for any baseline characteristics. BCVA: best-corrected visual acuity, DME: diabetic macular edema, and AMD: age-related macular degeneration.

**Table 2:** Outcome variables stratified by intraretinal fluid (IRF) vs. control and subretinal fluid (SRF) vs. control. Odds ratio (OR), 95% confidence interval, and *p* value listed for each outcome variable.

	IRF			SRF		
	Odds ratio	95% confidence	*p* value	Odds ratio	95% confidence	*p* value
*Age*	0.93	(0.89, 0.98)	0.01^*∗*^	1.12	(1.04, 1.21)	0.01^*∗*^

*Gender*	0.84	(0.29, 2.41)	0.74	1.40	(0.39, 5.07)	0.61

*Race*						
White	0.18	(0.04, 0.84)	0.03^*∗*^	1.87	(0.21, 16.7)	0.58
Black	0.60	(0.05, 6.80)	0.60	—	—	—

*Asian*						
Reading speed (s)	0.99	(0.98, 1.01)	0.68	1.00	(0.99, 1.02)	0.66
Contrast sensitivity (logcontrast)	0.02	(0.00, 0.46)	0.02^*∗*^	0.01	(0.00, 0.56)	0.02^*∗*^
Percent correct	0.98	(0.96, 1.01)	0.22	0.99	(0.96, 1.02)	0.51
Edit distance	1.09	(0.62, 1.90)	0.77	1.07	(0.58, 2.05)	0.78

^*∗*^ *p* value of statistical significance (<0.05). Visual acuity for all groups was measured at near distance. Control patients were blurred at near distance.

**Table 3:** Logistic regression analysis for base models assuming intraretinal fluid and subretinal fluid as the dependent variable. Average edit distance and intraretinal fluid were then added to the base equation. Nagelkerke *R*^2^ in this analysis assumes a “goodness of fit” for each model. Chi-square analysis and *p* value were calculated between each model.

	Df	−log likelihood	Nagelkerke *R*^2^	*χ* ^2^	*p* value
*Model A (IRF)*					
Base model A	3	66.89	0.26	—	—
Base model A + avg. edit distance	4	66.82	0.26	0.07	0.79
Base model A + % correct	4	66.21	0.27	0.68	0.41

*Model B (SRF)*					
Base model B	3	59.76	0.17	—	—
Base model B + avg. edit distance	4	59.61	0.17	0.15	0.70
Base model B + % correct	4	59.73	0.17	0.02	0.88

## Data Availability

The data used to support the findings of this study are available from the corresponding author upon request.

## References

[1] von Ahn L., Blum M., Hopper N. J., Langford J. (2003). CAPTCHA: using hard AI problems for security. *Lecture Notes in Computer Science*.

[2] von Ahn L., Maurer B., McMillen C., Abraham D., Blum M. (2008). reCAPTCHA: human-based character recognition via web security measures. *Science*.

[3] Mangione C. M., Phillips R. S., Seddon J. M. (1992). Development of the activities of daily vision scale. *Medical Care*.

[4] Kington R., Rogowski J., Lillard L., Lee P. P. (1997). Functional associations of “trouble seeing”. *Journal of General Internal Medicine*.

[5] Lundstrom M., Fregell G., Sjoblom A. (1994). Vision related daily life problems in patients waiting for a cataract extraction. *British Journal of Ophthalmology*.

[6] Javitt J. C., Brenner M. H., Curbow B., Legro M. W., Street D. A. (1993). Outcomes of cataract surgery. *Archives of Ophthalmology*.

[7] Mangione C. M., Lee P. P., Pitts J. (1998). Psychometric properties of the national eye Institute visual function questionnaire (NEI-VFQ). *Archives of Ophthalmology*.

[8] Lau J., Michon J. J., Chan W.-S., Ellwein L. B. (2002). Visual acuity and quality of life outcomes in cataract surgery patients in Hong Kong. *British Journal of Ophthalmology*.

[9] Lamoureux E. L., Maxwell R. M., Marella M., Dirani M., Fenwick E., Guymer R. H. (2001). The longitudinal impact of macular telangiectasia type 2 on vision-related quality of life. *Investigative Opthalmology & Visual Science*.

[10] Elliott D. B, Hurst M. A, Weatherill J. (1990). Comparing clinical tests of visual function in cataract with the Patient’s perceived visual disability. *Eye*.

[11] Hazel C. A., Petre K. L., Armstrong R. A., Benson M. T., Frost N. A. (2000). Visual function and subjective quality of life compared in subjects with acquired macular disease. *Investigative Opthalmology & Visual Science*.

[12] Issa P. C., Holz F. G., Scholl H. P. N. (2009). Metamorphopsia in patients with macular telangiectasia type 2. *Documenta Ophthalmologica*.

[13] Holman J., Lazar J., Feng J. H., D’Arcy J. Developing CAPTCHA for blind users.

[14] Baker C. W., Almukhtar T., Bressler N. M. (2013). Macular edema after cataract surgery in eyes without preoperative central-involved diabetic macular edema. *JAMA Ophthalmology*.

[15] Perente I., Alkin Z., Ozkaya A. (2014). Focal laser photocoagulation in non-center involving diabetic macular edema. *Medical Hypothesis, Discovery & Innovation Ophthalmology Journal*.

[16] von Ahn L., Blum M., Hopper N. J., Langford J. (2003). CAPTCHA: using hard AI problems for security. *Advances in Cryptology, Lecture Notes in Computer Science*.

[17] Wang S.-Y., Bentley J. L. CAPTCHA challenge tradeoffs: Familiarity of strings versus degradation of images.

[18] de Jong P. T. V. M. (2006). Age-related macular degeneration. *New England Journal of Medicine*.

[19] Usage statistics for CAPTCHA, https://www.drupal.org/project/usage/captcha

[20] Browning D. J., Altaweel M. M., Bressler N. M., Bressler S. B., Scott I. U. (2008). Diabetic Macular Edema: what is focal and what is diffuse?. *American Journal of Ophthalmology*.

[21] Young R. W. (1987). Pathophysiology of age-related macular degeneration. *Survey of Ophthalmology*.

[22] Evans J. M. (2006). Standards for visual acuity.

[23] Rabin J. (1994). Luminance effects on visual acuity and small letter contrast sensitivity. *Optometry and Vision Science*.

[24] Rassow B. (1999). Influence of luminance on contrast and glare sensitivity in the mesopic region. *Clinical Monthly Sheets for Ophthalmology*.

[25] Arend O., Remky A., Evans D., Stüber R., Harris A. (1997). Contrast sensitivity loss is coupled with capillary dropout in patients with diabetes. *Investigative Opthalmology & Visual Science*.

[26] Richman J., Spaeth G. L., Wirostko B. (2017). Contrast sensitivity basics and a critique of currently available tests. *Journal of Cataract and Refractive Surgery*.

[27] García A. S., Gómez A. G., Figueroa-Ortiz L. C., García-Ben A., García-Campos J. (2014). Relación entre el test de sensibilidad al contraste y el nivel de gravedad en pacientes con esclerosis múltiple. *Archivos de la Sociedad Española de Oftalmología*.

[28] Legault I., Gagné J.-P., Rhoualem W., Anderson-Gosselin P. (2010). The effects of blurred vision on auditory-visual speech perception in younger and older adults. *International Journal of Audiology*.

[29] Bertone A., Bettinelli L., Faubert J. (2007). The impact of blurred vision on cognitive assessment. *Journal of Clinical and Experimental Neuropsychology*.

